# Continuous autonomic system monitoring during neurosurgical procedures –proof of concept

**DOI:** 10.1007/s10877-025-01386-9

**Published:** 2025-11-29

**Authors:** Julian Zipfel, Dimitar Stoyanov, Marek Czosnyka, Berthold Drexler, Martin U. Schuhmann

**Affiliations:** 1 Department of Neurosurgery, Section of Pediatric Neurosurgery, University Hospital Tuebingen, Tuebingen, Baden-Württemberg 72076, Germany; 2 Brain Physics Laboratory, Division of Neurosurgery, Department of Clinical Neurosciences, Addenbrooke’s Hospital, University of Cambridge, Cambridge Biomedical Campus, Cambridge, UK; 3 Department of Anesthesiology and Intensive Care, University Hospital, Tuebingen, Germany; 4 Department of Neurosurgery, University Hospital Tuebingen, Hoppe-Seyler-Str. 3, 72076 Tuebingen, Germany

**Keywords:** Neuromonitoring, Autonomic system monitoring, Neurosurgery, Neuroanesthesia

## Abstract

Vegetative reactions are common during neurosurgical procedures. Known effects are mainly cardiovascular, including tachy- and bradyarrhythmia, hyper- and hypotonia as well as cardiac arrest. Computer-assisted real-time analysis of heart rate variability (HRV), baroreflex-sensitivity (BRS) allows for continuous evaluation of the autonomic nervous system (ANS). We analyzed ANS parameters during intracranial neurosurgical procedures. In this pilot study, we aim to provide proof-of-concept that ANS monitoring during surgery is feasible and yields stable results.We included 129 consecutive patients undergoing neurosurgery for intracranial pathologies over a period of four months. Heart rate (HR) and mean arterial pressure (MAP) were continuously monitored during routine anesthesiology care. Data were recorded via ICM + software. HRV, BRS and other vegetative parameters were calculated continuously. Intraoperative events such as hypo-/hypertonia or brady-/tachycardia were monitored.Mean age was 47.2 ± 17.7 years. Of all patients, 54.3% were male (*n* = 70). For every patient, four intraoperative episodes were defined: start of anesthesia until incision – start of incision until craniotomy – craniotomy until end of resection or intracranial manipulation – end phase until skin closure. BRS continuously decreased during cranial surgery, indicating stabilized autonomic function. Furthermore, blood pressure variability was increased during semi-sitting surgery.Autonomic system monitoring during neurosurgical procedures is safe and feasible. Intraoperatively, an increasing sympathetic activity has been observed without clear disctinction between surgical or anesthesiological events as underlying cause. Monitoring results are reproducible and may be of importance for the detection and prevention of intraoperative cardiovascular events.

**Supplementary Information:**

The online version contains supplementary material available at 10.1007/s10877-025-01386-9.

## Introduction

Brainstem and adjacent regions like the cerebellopontine angle are predilection sites for neurooncological and neurovascular diseases. Commonly encountered tumors in adults comprise vestibular schwannomas, gliomas, and meningiomas. In children, medulloblastomas, pilocytic astrocytomas, and ependymomas are the most common tumors around this location. Vascular lesions include cavernomas, arteriovenous malformations or neurovascular compression syndromes such as hemifacial spasm, and trigeminal neuralgia. Naturally, neurosurgical interventions in this region are accompanied by significant risks of complications.

In addition to direct damage to neuronal, axonal, and neurovascular structures that might result in neurological deficits, vegetative or autonomic reactions are common and undesired phenomena during surgery around these sensitive locations. Known effects include tachy- and bradyarrhythmia, hyper- and hypotonia as well as cardiac arrest.[1] More rarely, Takotsubo syndrome has been reported, probably due to trigeminocardiac reflex [2, 3], vagal affection, or baroreceptor stimulation [4]. Trigeminocardiac reflex is common in otolaryngologic and maxillofacial surgeries but may also be encountered in numerous neurosurgical operations [5] especially anterior transpetrosal [6], retrosigmoid [7] and transsphenoidal [8] approaches.

Systematic monitoring of vegetative functions of the autonomic nervous system (ANS) during neurosurgical procedures is not routinely performed. Commonly monitored parameters during general anesthesia for intracranial surgery are heart rate, invasive blood pressure, electrocardiography, peripheral oxygen saturation, as well as respiratory parameters. Vegetative monitoring parameters include heart rate variability (HRV) and baroreflex-sensitivity (BRS). HRV is calculated via the standard deviation between cardiac-cycle intervals [9] whereas BRS evaluates baroreceptor reflex via a cross-correlation after adjusting for variability of the delay between systolic blood pressure and RR-interval (time elapsed between two successive R-waves) [10]. Computerized monitoring and second-to-second calculation of these parameters (although with overlapping time-windows) allows for continuous real-time evaluation of the ANS [11]. 

Vegetative monitoring has been proposed to have utility in cases of severe traumatic brain injury (TBI) as well as subarachnoid hemorrhage [12]. The well-known Cushing reflex describes hypertension, bradycardia and respiratory changes in patients with elevated intracranial pressure (ICP), associated with suppression of parasympathetic activity [13]. Injury of the right hemisphere in neonates correlates with increased HRV, whereas left-sided injury might suppress HRV [14]. 

In acute cerebral injury of different etiology, suppressed HRV and BRS are associated with higher morbidity and mortality [12]. This effect seems to be independent of other variables such as increased intracranial pressure [15]. 

Other fields of interest for vegetative monitoring include Parkinson’s disease, wherein reduced HRV is found in association with neurodegeneration in the region of the medulla oblongata [16]. Furthermore, MRI studies found suppressed parasympathetic activity in the locus coeruleus, correlating with lowered high-frequency-HRV [17]. 

Brain stem lesions may lower HRV [18], because normally adequate HRV and blood pressure variability depends on intact brainstem function [19]. Cases of persistent hypertonia and postoperative cardiovascular instability are observations that are not uncommon after surgery around the brainstem [20, 21]. However, standardized criteria and monitoring methods are not available in every institution [22]. 

The proposed utility of our ANS monitoring system is to create a feedback mechanism for the relevant brainstem functions which are not part of routine intraoperative neurophysiologic monitoring. Our hypothesis was that intraoperative vegetative monitoring may be able to predict intra- and postoperative cardiovascular events. Through correlation of monitoring parameters with intraoperative events of a cardiovascular nature, we want to establish an early detection system for critical cardiovascular reactions that are attributable to surgical manipulation. In this pilot study, we aim to provide proof-of-concept that ANS monitoring during neurosurgical procedures is feasible and yields consistent results.

## Materials and methods

Following institutional review board approval (627/2018BO1), we performed the study and analyzed the results. Inclusion criteria were as follows: plan open neurosurgical procedure via craniotomy or transsphenoidal approach with the need of continuous invasive arterial blood pressure measurement.

With a single monitoring setup and single examining scientist setup, the operating theatre with the most relevant case mix (with a focus on pathologies with brain stem or cranial nerve association and planned craniotomy) for the day was chosen. Patients were screened and asked for consent the day before surgery. Four patients did not consent to the study. We prospectively included 129 consecutive patients undergoing intracranial surgery over a time period of 4 months. No other patients were excluded from further analysis.

Patients eligible for inclusion or their legal guardian had to give formal written consent to the data collection.

Inclusion criteria were as follows: patient was admitted for elective cranial surgery and informed consent was obtained. All parameters directly measured (heart rate, invasive blood pressure via arterial line, peripheral oxygen saturation) were monitored via monitoring devices (IntelliVue AD75, Philips, Amsterdam, Netherlands) for anesthesiologic live-evaluation as per routine. A digital output connected to a stand-alone research computer allowed for sampling of monitoring parameters at 100 Hz via ICM + software (Cambridge Enterprise Ltd, Cambridge, UK). ANS parameters were calculated as described below and continuously updated to form time-trends. relative and absolute value of high frequency heart rate.

### Baroreceptor sensitivity (BRS, ms/mmHg)

BRS was calculated via modification of the sequential cross-correlation method, describing the magnitude of response in the heart-beat interval to a change in blood pressure described by Westerhof et al. [23] The modified function applying an automated detection algorithm uses arterial blood pressure systolic peaks to create RR intervals time series. The slope of the linear regression between 10 s series of RR intervals and the corresponding 10 s series of systolic blood pressure is then calculated. In order to remove the influence of an unknown time delay of the baroreceptor response, cross-correlation is used to maximize the correlation coefficient. The RR window is shifted against the systolic pressure window in a stepwise manner and the highest valid correlation is reported. In order to ensure that the correlation calculations are always performed on the same number of data points irrespective of the lag applied to RR series, the actual data buffer is extended with each window shift. Valid BRS value is returned only if the correlation coefficient is significant at *p* < 0.01, and if no ectopic beats (irregular beats) are detected by the software. To compensate for the influence of uncorrelated noise the slope returned is divided by the correlation coefficient. The BRS value is updated every 10 s and expressed as median in ms/mmHg.

### Heart rate variability (HRV)

In the frequency domain we calculated frequency-specific indexes: High-Frequency component (HF; Power Spectral Density of the beat-to-beat interval time series that returns high-frequency band component 0.15–0.4 Hz), Low Frequency component (LF; 0.004–0.15 Hz) and the ratio between the two (LF/HF ratio). Normalized power is calculated as a ratio of the components power divided by the total power minus VLF power (very low frequency < 0.004 Hz, expressed in %). Relative power is calculated as a ratio of the component’s power divided by the sum of HF, LF and VLF components total power (expressed in %) [24]. Additional parameters are: HRhfRel: relative value of high frequency heart rate, HRVhf/lfhf: heart rate variability low frequency/high frequency.

### Mean arterial pressure (MAP, mmHg)

MAP-derived parameters were also calculated as the high-frequency component (HF) via MAP high-frequency signal power (0.15–0.4 Hz). Five-minute-long epochs were used. MAP low-frequency signal power (LF) is in the specified range of 0.04–0.15 Hz.

MAP_s was calculated as MAP pulse variability measures (systolic pressure was used). Its function is calculating systolic arterial pressure pulse metrics and returning its variability values.

Predefined routine surgical steps like skin incision, craniotomy, durotomy etc. were documented in the electronic anaesthesia protocol (ICCA, Philips Medical Systems, Andover, MA) and used for the definition of different procedural episodes. These were defined as baseline (start of anesthesia until incision as episode 1), surgery begin (start of incision until craniotomy as episode 2), manipulation (craniotomy until end of resection/intracranial manipulation as episode 3) and end (dura closure to end of surgery as episode 4).

Anesthesia was standardized and performed as TIVA (total intravenous anesthesia) with the following parameters: 5 mg/kg/h of propofol, 0,5 µg/kg/min remifentanil, crystalloids and norepinephrine by peripheral intravenous line to achieve a MAP goal of ~ 80mmHg, end-tidal carbon dioxide (etCO2) goal of ~ 35mmHg. Significant events such as such as hypo-/hypertension, asystole, brady- and tachykardia etc. were recorded and documented. Five-minute timeframes were manually removed after bolus administration of e.g. catecholamines or analgesics, change of anesthetic agent infusion rate or after arterial blood gas analysis to minimize artifacts. Events during these timeframes were excluded from further analysis.

Statistics were analyzed using SPSS Statistics 25 (IBM, NY, U.S.A.). Continuous data were presented as mean (± standard deviation), whereas categorical data were shown as percentages. Continuous variables were tested for equality of variances by Levene’s test. Normal distributed parametric variables with equal variances were compared using the unpaired or paired t-test, otherwise Mann-Whitney U test was performed. Nominal variables were tested with Fisher´s exact test. P values < 0.05 were regarded as significant.

## Results

### Basic patient information

Details are presented in Table 1. We included 129 consecutive patients undergoing neurosurgical procedures for intracranial pathologies. Median age was 48 years (range 1–87 years). Of all patients, 54.3% were male (*n* = 70). Underlying pathologies were mostly benign tumors (77.5%, *n* = 100), followed by malignant tumors (14.7%, *n* = 19) and vascular (7.8%, *n* = 10). Most interventions were for infratentorial pathologies (58.9%, *n* = 76), followed by supratentorial (34.9%, *n* = 45) and pituitary (6.2%, *n* = 8). Significant mass effect was seen in preoperative imaging (e.g. edema, compression of ventricles, midline-shift) in 49.6% of cases (minor *n* = 27 without consequent neurological impairment, major *n* = 37 with resulting neurological impairment). Hydrocephalus was present in 7 cases (5.4%). Tumor association to the tentorium was present in 6 cases (4.7%). Association, i.e. displacement of cranial nerves was seen in 98 cases (76.0%).

Length of the individual episodes 1 through 4 was 10.1+/−0.1 min, 64.9+/−27.2 min, 72.4+/−65.0 min and 49.8+/−21.5 min, respectively.

**Table 1:** Basic patient and surgery parameters

age	47.2 ± 17.7 years						
sex	45.7% female (*n* = 59)	54.3% male (*n* = 70)					
pathology	14.7% malignant tumor (*n* = 19)	77.5% benign tumor (*n* = 100)	7.8% vascular (*n* = 10)				
pathology location	58.9% infratentorial (*n* = 76)	34.9% supratentorial (*n* = 45)	6.2% pituitary (*n* = 8)				
mass effect	50.4% no (*n* = 65)	20.9% minor (*n* = 27)	28.7% major (*n* = 37)				
hydrocephalus	94.6% no (*n* = 122)	5.4% yes (*n* = 7)					
association to tentorium	95.3% no (*n* = 123)	4.7% yes (*n* = 6)					
association with cranial nerves	76% yes (*n* = 98)	24% no (*n* = 31)					
	9.3% optic (*n* = 12)	3.1% 3rd (*n* = 4)	9.3% 5th (*n* = 12)	34.9% 7th (*n* = 45)	34.1% 8th (*n* = 44)	3.9% 9th/10th/12th (*n* = 5)	0.8% 11th (*n* = 1)
event	53.5% yes (*n* = 69)	46.5% no (*n* = 60)					
	36.4% bradycardia (*n* = 47)	11.6% tachycardia (*n* = 15)	25.6% hypotonia (*n* = 33)	29.5% hypertonia (*n* = 38)			

### Intraoperative events

**Fig. 1:**
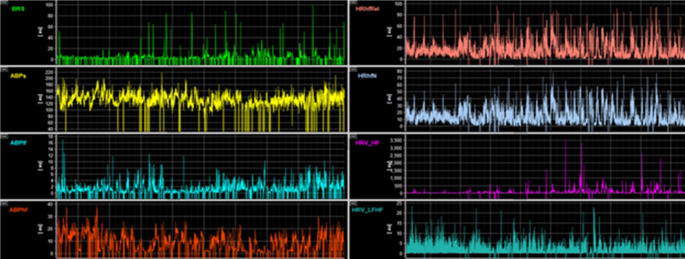
Exemplary excerpt of several monitoring parameters of a single patient

Concerning intraoperative events (> 1 min), we saw 47 cases of pronounced bradycardia/asystole (heart rate below 50/s, 36.4%), 15x tachycardia (heart rate above 120/s, 11.6%), 33x hypotension (systolic blood pressure lower than 80mmHg, 25.6%) and 38x hypertonia (systolic blood pressure higher than 130mmHg, 29.5%). MAP_s was seen to be significantly higher in males during monitoring episodes 1 through 3 (female vs. male 1: 109.7 ± 13.6 vs. 115.7 ± 12.5, *p* = 0.013, 2: 114.1 ± 11.3 vs. 120.3 ± 12.5 *p* = 0.004, 3: 117.0 ± 11.9 vs. 122.0 ± 12.0 *p* = 0.023, 4: 79.1 ± 5.9 vs. 78.3 ± 4.5 *p* = 0.370). Figure 1 provides an example of continuous autonomic system monitoring via ICM+.

BRS Baroreflex sensitivity, ABPs/lf/hf: arterial blood pressure systolic/low frequency/high frequency, HRhfRel/N: relative and absolute value of high frequency heart rate, HRVhf/lfhf: heart rate variability low frequency/high frequency.

Patients with significant episodes of bradycardia or asystole unsurprisingly had significantly lower mean HR during monitoring episodes 2 through 4 (2: *p* = 0.014 54.0 ± 9.6 vs. 60.2 ± 18.6; 3: *p* = 0.003 58.6 ± 12.0 vs. 67.0 ± 18.7; 4: *p* = 0.008 60.7 ± 12.8 vs. 67.9 ± 17.1). Furthermore, MAP_s (episode 2) as well as MAPlf (episode 3) were lower (*p* = 0.018 115.5 ± 11.9 vs. 120.9 ± 12.5; *p* = 0.012 2.4 ± 2.1 vs. 4.0 ± 5.2). Patients with significant episodes of tachycardia had significantly lower HRhf_rel (episode1) and HRhf_rel (episode 2) (*p* = 0.005 34.8 ± 18.5 vs. 49.9 ± 22.6, *p* = 0.020 34.6 ± 16.5 vs. 45.4 ± 18.8) as well as lower MAP (episode 4) (*p* = 0.013, 78.3 ± 5.0 vs. 81.8 ± 5.9).

### Monitoring parameter dynamics

**Fig. 2:**
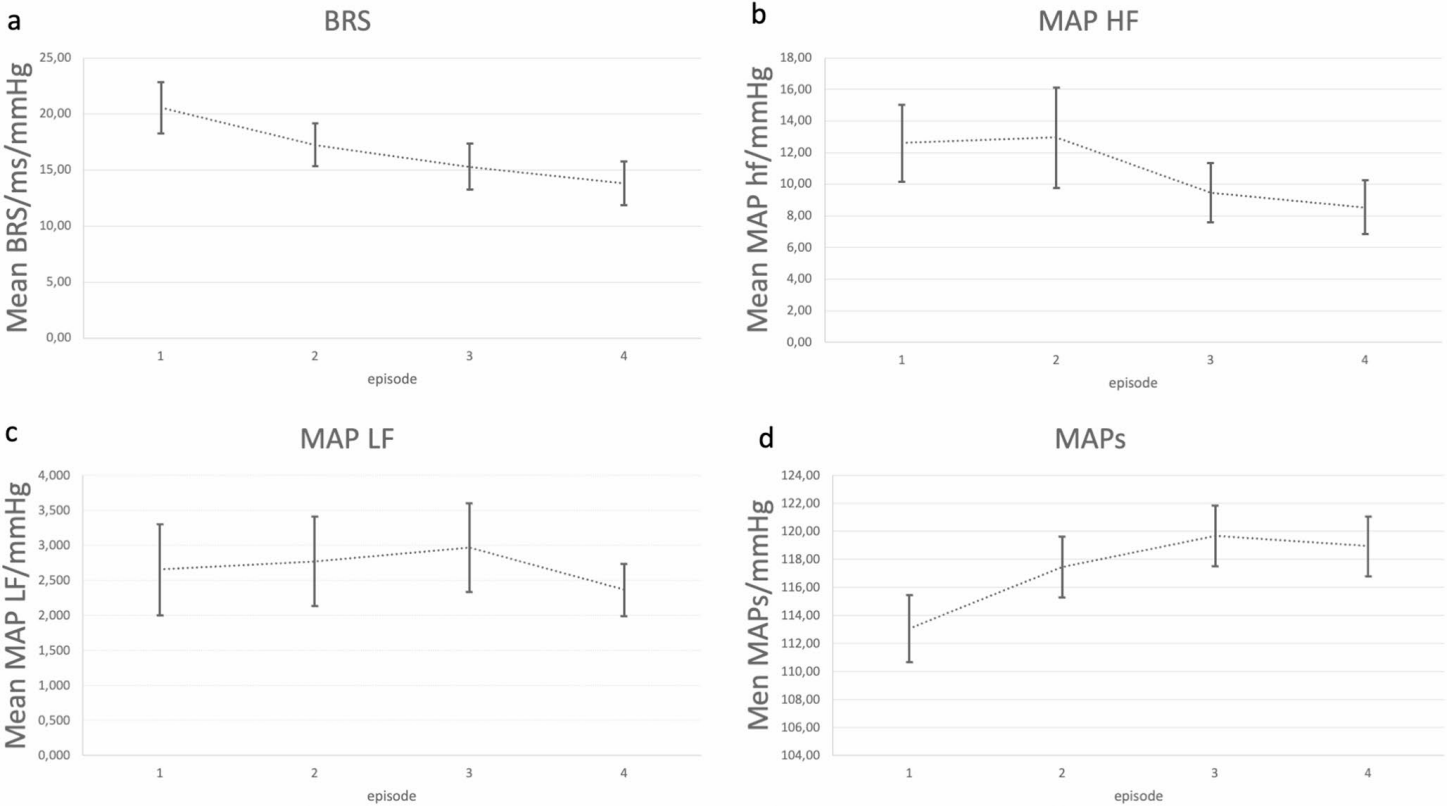
ANS parameter dynamics in the whole cohort

Table 2; Fig. 2 show the comparative monitoring results of the whole cohort (*n* = 129) during the different monitoring episodes.

**Table 2:** Comparison of ANS parameter dynamics during episodes 1 through 4 in the whole cohort (*n* = 129)

	1	2	3	4	1 vs. 2	1 vs. 3	1 vs. 4	2 vs. 3	2 vs. 4	3 vs. 4
	mean ± SD				*p*-value					
MAP (mmHg)	78.2 ± 8.2	79.4 ± 6.3	80.1 ± 5.7	78.8 ± 5.2	0.030	0.006	0.448	0.139	0.152	0.004
BRS (ms/mmHg)	20.7 ± 12.0	16.7 ± 10.2	14.4 ± 10.7	13.4 ± 9.7	< 0.001	< 0.001	< 0.001	0.001	< 0.001	0.023
HR (1/min)	51.7 ± 12.6	56.5 ± 14.2	62.0 ± 15.9	63.3 ± 15.4	< 0.001	< 0.001	< 0.001	< 0.001	< 0.001	0.001
HR hf rel	36.7 ± 19.6	35.6 ± 17.0	33.1 ± 18.33	34.0 ± 16.0	0.420	0.068	0.183	0.091	0.253	0.248
HRV lfhf	1.44 ± 3.05	1.14 ± 0.97	1.25 ± 0.73	1.24 ± 0.72	0.309	0.463	0.465	0.190	0.315	0.452
MAP hf (mmHg)	12.6 ± 12.5	12.2 ± 11.7	7.8 ± 8.8	7.1 ± 7.3	0.005	< 0.001	< 0.001	0.001	0.001	0.087
MAP lf (mmHg)	2.65 ± 3.35	2.76 ± 3.70	2.93 ± 3.74	2.28 ± 1.94	0.578	0.529	0.294	0.563	0.171	0.029
MAP_s (mmHg)	113.2 ± 13.3	117.0 ± 12.3	119.4 ± 12.2	118.6 ± 12.0	< 0.001	< 0.001	< 0.001	0.324	0.123	0.296
BRS r (ms/mmHg)	0.528 ± 0.099	0.523 ± 0.070	0.525 ± 0.087	0.523 ± 0.085	0.578	0.903	0.562	0.839	0.793	0.940

Episode 1 start of anesthesia until incision, Episode 2 start of incision until craniotomy, Episode 3 craniotomy until end of resection/intracranial manipulation, Episode 4 dura closure to end of surgery, MAP: mean arterial pressure, BRS: baroreceptor sensitivity, HR heart rate, hf: high frequency, lf: low frequency, HRV: heart rate variability, MAP_s: MAP pulse variability measure

Graphs of ANS mean parameter dynamics between the four episodes 1 baseline (start of anesthesia until incision), 2 surgery begin (start of incision until craniotomy), 3 manipulation (craniotomy until end of resection/intracranial manipulation) and 4 end (dura closure to end of surgery) (a) BRS baroreceptor reflex sensitivity in ms/mmHg, (b) MAPhf high frequency MAP in mmHg (c) MAPlf low frequency MAP in mmHg (d) MAP_s in mmHg; error bar: standard error.

**Fig. 3:**
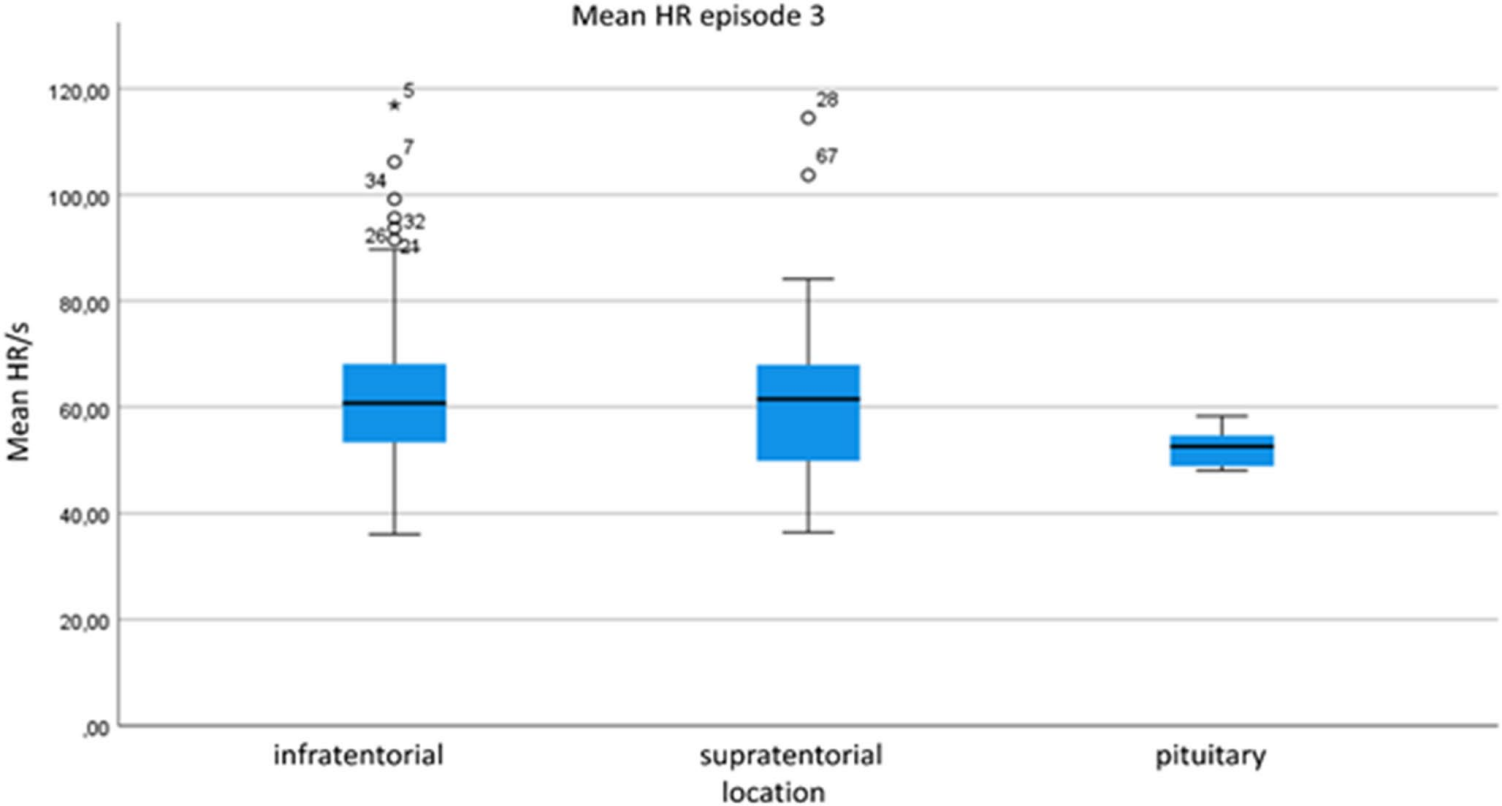
Comparison of heart rate during episode 3 in infra-/supratentorial craniotomy and transsphenoidal surgery

During surgery for infratentorial pathologies, MAP was significantly higher than for supratentorial pathologies during episodes 2, 3 and 4 (*p* = 0.001, *p* = 0.029, *p* = 0.001). Furthermore, MAP_s were significantly higher during episodes 2, 3, and 4 (*p* = 0.029, p = < 0.001, *p* = 0.014). This was also true when comparing against pituitary (*p* = 0.001). HR was significantly lower in infratentorial vs. pituitary, *p* < 0.001 and higher in supra-tentorial vs. pituitary (*p* = 0.003) during episode 3 (Fig 3). BRS was significantly higher in infra- and lower in supra- as compared to pituitary during episode 3 (infra- vs. pituitary *p* = 0.019, supra- vs. pituitary *p* = 0.005). HR was significantly lower during episode 4 in pituitary (infra- vs. pituitary *p* = 0.001, supra- vs. pituitary *p* = 0.002). Table 3. gives the mean monitoring results for the different episodes.

**Table 3:** Comparison of monitoring parameters in different surgical locations

	monitoring episode	infratentorial		supratentorial		pituitary	
		mean	SD	mean	SD	mean	SD
MAP (mmHg)	1	78,61	8,40	76,87	7,75	81,74	7,72
	2	80,97	5,67	76,86	6,18	79,26	7,66
	3	82,30	5,21	76,61	4,81	78,67	5,56
	4	80,13	5,20	76,63	4,56	76,70	4,62
BRS (ms/mmHg)	1	21,66	11,90	18,72	11,42	20,05	15,70
	2	18,59	11,89	15,43	8,03	14,62	8,79
	3	15,65	10,95	14,88	11,61	13,64	10,72
	4	13,39	9,77	13,94	11,61	18,27	16,23
HR (1/min)	1	50,97	13,27	53,14	12,44	49,52	6,30
	2	55,63	14,76	57,40	13,17	54,75	5,38
	3	62,90	15,98	61,61	15,16	52,38	3,63
	4	63,86	15,52	64,18	14,31	52,24	5,48
HR_hf_rel	1	37,42	18,84	36,16	19,61	31,80	27,26
	2	38,05	16,36	33,77	17,69	27,56	17,62
	3	31,89	17,12	33,04	18,85	40,34	24,12
	4	34,86	16,42	32,45	15,15	36,45	18,13
HRV_lfhf	1	1,43	3,32	1,46	2,84	1,28	0,71
	2	1,13	1,12	1,10	0,60	1,16	0,72
	3	1,28	0,75	1,33	1,10	1,02	0,63
	4	1,16	0,73	1,28	0,57	1,50	1,14
MAP_hf (mmHg)	1	14,44	13,85	9,55	10,11	14,07	11,79
	2	15,79	21,63	9,19	11,46	7,27	5,50
	3	9,88	10,89	9,31	11,11	6,38	4,56
	4	8,73	10,38	8,16	8,74	9,12	9,04
MAP_lf (mmHg)	1	2,77	4,07	2,26	1,91	3,70	3,03
	2	3,00	4,32	2,21	2,35	3,76	2,24
	3	3,41	4,32	2,02	1,89	4,22	2,96
	4	2,41	2,18	2,17	2,06	3,01	1,92
MAP_s (mmHg)	1	113,54	11,50	111,86	16,43	115,26	10,35
	2	120,10	10,93	114,15	13,24	111,39	14,11
	3	123,80	10,65	115,03	12,04	108,86	10,81
	4	121,77	11,28	115,46	12,19	111,18	11,20

Episode 1 start of anesthesia until incision, Episode 2 start of incision until craniotomy, Episode 3 craniotomy until end of resection/intracranial manipulation, Episode 4 dura closure to end of surgery, MAP: mean arterial pressure, BRS: baroreceptor sensitivity, HR heart rate, hf: high frequency, lf: low frequency, HRV: heart rate variability, MAP_s: MAP pulse variability measure

### Positioning

In total, 99 patients were positioned supine and 30 semi-sitting. Comparing these groups, we found significantly higher MAP during episode 1 in the supine group (*p* = 0.027 79.1+/−8.3 vs. 75.3+/−7.0). MAPhf was lower in episode 2 (*p* = 0.007 10.6+/−15.6 vs. 20.8+/−23.8). BRS was lower both in episodes 2 and 3 (2: *p* = 0.019 16.0+/−10.1 vs. 21.5+/−11.1; 3: *p* = 0.033 14.0+/−10.8 vs. 19.2+/−11.2). HR was higher in episode 2 (*p* = 0.031 57.6+/−14.6 vs. 51.5+/−9.0). When looking at MAP_s, we found no difference during episode 1 but significantly lower values in supine positioning during episodes 2 through 4 (1: *p* = 0.968 113.1+/−14.0 vs. 113.0+/−10.7; 2: *p* = 0.002 115.6+/−11.7 vs. 123.4+/−12.7; 3: *p* < 0.001 116.9+/−11.9 vs. 129.0+/−7.9; 4: *p* < 0.001 116.1+/−11.5 vs. 128.0+/−9.0).

### Monitoring parameters associated with clinical indices

Table 4 summarizes significant findings of ANS monitoring associated with clinical indices. No significant differences could be found between patients with and without hydrocephalus as well as with or without association to tentorium or trigeminal, lower cranial or accessory nerve.

**Table 4:** ANS finding associated with clinical indices

	parameter	Monitoring episode	yes	no	*p*-value
Malignant tumor?	HR (1/s)	1	61.3 ± 20.1	50.1 ± 10.1	0.001
		2	68.5 ± 21.1	54.7/−10.8	< 0.001
		3	73.9 ± 22.9	60.1 ± 12.6	< 0.001
		4	72.4 ± 21.7	62.2+/13.0	0.007
Post fossa mass effect?	MAP hf (mmHg)	2	27.8 ± 49.6	12.2 ± 15.4	0.001
	BRS (ms/mmHg)	4	20.3 ± 27.6	13.6 ± 9.9	0.037
	MAP (mmHg)	3	76.4 ± 4.7	80.3 ± 5.7	0.006
	MAP_s (mmHg)	3	106.9 ± 10.5	120.2 ± 12.0	0.005
		4	107.4 ± 12.5	119.4 ± 11.8	0.023
Cranial nerve association?	MAP hf (mmHg)	1	14.8 ± 13.4	6.0 ± 6.2	0.002
	BRS (ms/mmHg)	2	18.5 ± 11.0	13.2 ± 8.0	0.021
		3	16.6 ± 11.3	11.2 ± 9.4	0.027
	MAP (mmHg)	3	81.1 ± 5.6	76.9 ± 5.0	< 0.001
2nd nerve affection	HR (1/s)	3	52.8 ± 4.8	62.7 ± 15.8	0.033
		4	112.1 ± 10.5	120.5 ± 12.1	0.022
	MAP_s (mmHg)	3	53.2 ± 6.0	64.3 ± 15.1	0.018
3rd nerve affection	MAP_s (mmHg)	4	106.3 ± 8.4	119.3 ± 11.9	0.032
7th nerve affection	BRS (ms/mmHg)	1	23.0 ± 12.0	18.7 ± 11.6	0.032
		2	21.6 ± 11.5	15.0 ± 79.4	0.001
	MAPhf (mmHg)	1	17.4 ± 14.9	10.2 ± 10.5	0.005
		2	20.3 ± 26.5	9.0 ± 9.8	0.001
	MAP_s (mmHg)	2	122.3 ± 11.4	114.9 ± 12.1	0.001
		3	126.7 ± 8.4	116.2 ± 12.3	< 0.001
		4	126.0 ± 9.4	115.1 ± 11.6	< 0.001
	MAP (mmHg)	3	81.7 ± 3.9	79.2 ± 6.4	0.015
		4	80.3 ± 4.6	77.8 ± 5.4	0.008
	HR (1/s)	1	47.5 ± 8.2	53.8 ± 14.0	0.009
		2	52.0 ± 8.6	58.4 ± 15.4	0.012
8th nerve affection	BRS (ms/mmHg)	1	23.9 ± 12.1	18.9 ± 11.6	0.040
		2	21.7 ± 11.6	15.0 ± 9.3	0.001
	MAPhf (mmHg)	1	17.1 ± 15.0	10.5 ± 10.6	0.011
		2	20.2 ± 26.8	9.2 ± 9.9	0.002
	MAP_s (mmHg)	2	122.8 ± 11.0	114.8 ± 12.1	< 0.001
		3	127.3 ± 7.5	115.9 ± 12.3	< 0.001
		4	126.5 ± 9.0	115.0 ± 11.6	< 0.001
	MAP (mmHg)	3	82.0 ± 3.4	79.0 ± 6.4	0.005
		4	80.6 ± 4.3	77.7 ± 5.4	0.003
	HR (1/s)	1	47.6 ± 8.3	53.6 ± 13.9	0.013
		2	52.2 ± 8.7	58.2 ± 15.4	0.018

Episode 1 start of anesthesia until incision, Episode 2 start of incision until craniotomy, Episode 3 craniotomy until end of resection/intracranial manipulation, Episode 4 dura closure to end of surgery, MAP: mean arterial pressure, BRS: baroreceptor sensitivity, HR heart rate, hf: high frequency, lf: low frequency, HRV: heart rate variability, MAP_s: MAP pulse variability measure

Mean heart rate (HR, 1/s), comparison of infratentorial, supratentorial and pituitary location of the operated lesion during episode 3 (craniotomy until end of resection/intracranial manipulation).

## Discussion

In our cohort of 129 consecutive patients undergoing cranial surgery, we were able to yield valid data on the ANS via real-time monitoring. The recorded data appear stable during the observed monitoring episodes.

### BRS/pain

We were able to demonstrate that BRS consistently decreased during general anesthesia for cranial surgery. BRS is a surrogate for the magnitude of the response of HR to changes in MAP and may interact with cerebral autoregulation.

No association between BRS and examined clinical indices was observed. Several hypotheses for our observation exist. The decrease of BRS in our cohort may be confounded by continuous ventilation, fluid management or use of catecholamines [25] during cranial surgery. Also, correlation of decreased BRS with increasing pain has been found [26] and may predict postoperative pain outcome [27]. ANS monitoring has come into focus as during general anesthesia, maintaining optimal analgesia may be challenging. Anesthetized patients cannot self-report pain or exhibit pain-related behaviors. Future prospective data may try to elicit the question of decreasing BRS during cranial surgery.

In addition to BRS, indices such as the Analgesia Nociception Index based on HRV have been promoted for assessment of intraoperative pain. The underlying assumption is that a nociceptive stimulus may lead to sympathetic response, increasing low frequency and decreasing high frequency domains [28]. In our cohort, MAP and MAPlf showed an initial increase until episode 3 with an eventual decrease. This effect was concomitant with a decrease of MAPhf in episode 3. Increasing sympathetic tone until episode 3 with decrease of MAPhf reflects parasympathetic inactivity whereas increasing MAPlf reflects sympathetic activity. Concomitantly, HR also increased during all episodes. Interpretation of these observations is limited as effects of surgery may not be distinguished from effects of anesthesia.

### Autonomic dysfunction

Autonomic dysfunction has been associated with poor outcomes in cases of subarachnoid hemorrhage [29]. Increased BRS in patients with cranial nerve affection during episodes 2 and 3 may be a sign of autonomic dysfunction, concomitant with parasympathetic activity as indicated by elevated MAPhf during episode 1 [24]. 

Patients with significant episodes of bradycardia or asystole, unsurprisingly had significantly lower mean HR during monitoring episodes 2 through 4. Furthermore, MAP during episode 2 as well as MAPlf during episode 3 were lower. BRS during episode 2 was also significantly lower. Patients with significant episodes of tachycardia had significantly lower HRhfrel during episode 1 and HRhfrel during episode 2 as well as lower MAP during episode 4.

### Patient positioning

MAP was shown to be lower in semi-sitting during episode 1, probably due to orthostatic effects. In succeeding episodes, no differences were seen. MAP_s on the other hand was significantly higher in semi-sitting positioning from episode 2 onwards. MAP_s is a pulse variability measure showing increased systolic blood pressure variability during semi-sitting positioning and thus indicating autonomic dysfunction [30]. This might be the consequence of increased doses of noradrenaline either due to patient positioning by itself or due to higher blood pressure targets for neurophysiology monitoring.

### Limitations

Limitations of our study arise from the mere observational methodology adopted. Data was collected prospectively without control group. No matching of groups was performed. While standardized anesthesia was utilized in all cases, no matching for medication and arterial hypertension was performed. Individual pharmacological data has not been considered in this dataset and further analysis should include information such as the opioid/propofol ratio and corresponding EEG effects. Also, the presented dataset is limited regarding possible underlying medical confounding factors, such as (neuro)vasculopathies, history of stroke, as these may signifcantly impact the autonomic nervous system.

Additionally, physiologic and pharmacologic mechanisms can have transient effects. For example, the baroreflex arc is important in the short-term regulation of the cardiovascular system and compensates for spontaneous fluctuations in blood pressure. Propofol-induced general anesthesia is also in fact a well-known depressor of autonomic nervous system and cardiac baroreflex control. During general anesthesia, BRS is often reduced [31–34]. Furthermore, HRV may be influenced by ventilation and medication [35] e.g. propofol decreases MAP and sympathetic nerve activity [36]. 

### Outlook

While general therapeutic approaches exist with the possibility of modulating blood pressure and respiratory parameters in an experimental deep brain stimulation setting [37]. Prevention of intraoperative autonomic dysregulation may be the best option for mitigating the known risks arising from vegetative reactions. In this experiential study, the prospect of our ANS monitoring system is to create a feedback mechanism for the relevant autonomic brainstem functions in addition to the routine cranial nerve neurophysiology and other monitoring like somatosensory and motor evoked potentials. Early identification and real-time monitoring of ANS may have tangible clinical utility and benefit.

## Conclusions

In this pilot study we were able to demonstrate that autonomic system monitoring during neurosurgical procedures is safe and feasible. Furthermore, it yields consistent and reliable results both intra- and inter- individually. The interpretation of these measurements and attribution to surgical or anesthesiological events is limited though. Increased sympathetic and decreased parasympathetic responses have been observed without clear disctinction between surgical or anesthesia as underlying cause. Monitoring results are reproducible and may be of importance for the detection and prevention of intraoperative cardiovascular events.

## Supplementary Information

Below is the link to the electronic supplementary material.

Supplementary Material 1

## Data Availability

Data is available from the corresponding author upon reasonable request.
